# Orbital venous-lymphatic malformation: Role of imaging

**DOI:** 10.4103/0974-620X.57316

**Published:** 2009

**Authors:** Anuj Mishra, Khalifa Alsawidi, Ramadan Abuhajar, Ehtuish F Ehtuish

**Affiliations:** Department of Radiology, National Organ Transplant Centre, Central Hospital, Tripoli, Libya; 1 Department of Ophthalmology, Eye Hospital, Tripoli, Libya; 2 AlKhoms Hospital, Alkhoms, Libya; 3 Department of Surgery, University of AlFateh, Tripoli, Libya

Hemangiomas and venous lymphatic malformations are the two most common orbital vascular lesions seen in pediatric patients. Orbital venous-lymphatic malformations (OVLMs) (previously referred to as ′lymphangiomas′) are uncommon, benign cystic type I vascular malformations.

OVLMs may remain clinically unapparent or might manifest in childhood with slowly progressive proptosis, periorbital swelling and displacement of globe. These malformations usually enlarge slowly. Spontaneous intraorbital hemorrhage or venous thrombosis may cause sudden acute proptosis, severe pain, compressive optic neuropathy or loss of vision, when intervention is indicated [Table 1]. Orbital venous-lymphatic malformations may be associated with conjunctival and episcleral involvement.[1] Lesions can extend to ipsilateral hard or soft palate and face. They may also be associated with noncontiguous intracranial vascular anomalies.[2,3]

**Table 1:** Indications for therapeutic interventions

progressive proptosis
Sudden increase of proptosis
Loss of vision
Severe periorbital pain
Presence of superficial mass

**Figure 1:**
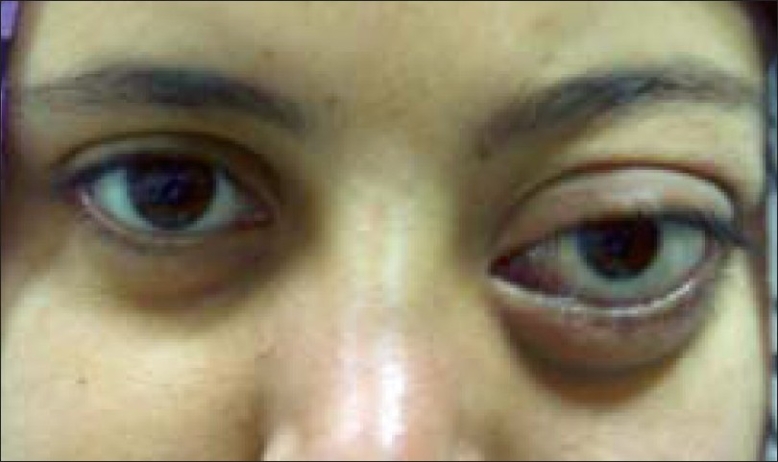
Patient with left proptosis

**Figure 2:**
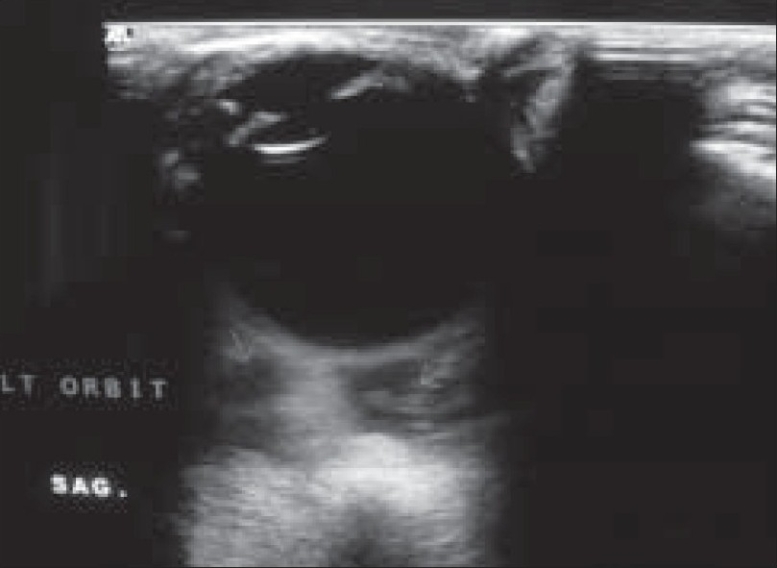
B-mode ultrasonography showed retrobulbar (intraconal) cystic lesions (Arrows)

**Figure 3:**
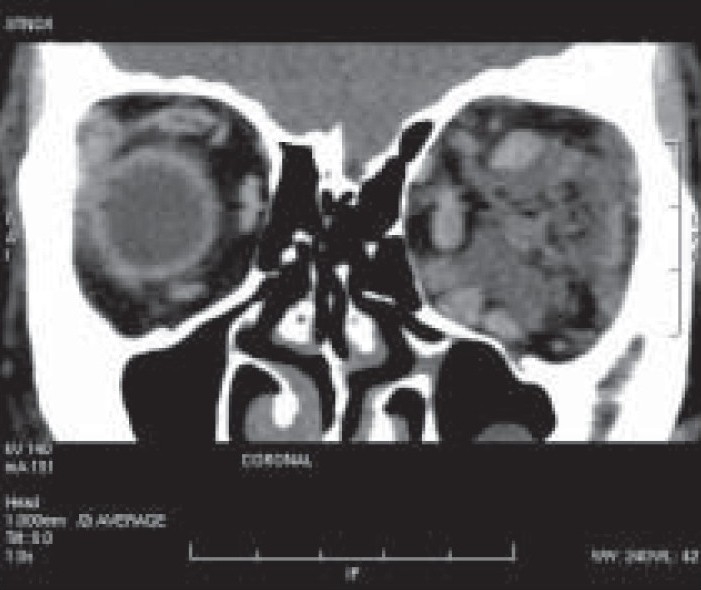
Reformatted coronal post-contrast thin section CT scan shows the retrobulbar unencapsulated non-enhancing intraconal lesions

A 20-year-old girl presented with left-sided progressive painless orbital proptosis since birth [Figure 1]. On examination, the visual acuity was 6/6 in the right eye (OD) and 6/36 in the left eye (OS). Intraocular pressures were 12 mmHg OD and 15 mmHg OS. External examination revealed proptosis on the left with an exophthalmometry (Hertel) reading of 18 mm OD and 26 mm OS at a base of 110 mm. Lid examination was normal OD but revealed a moderate ptosis OS (MRD = 1 mm; normal levator function). The pupils were round, equal, briskly reactive and without a relative afferent pupillary defect. Extraocular motility and confrontational visual fields were full. Although there was no diplopia, there was a mild limitation of the motility in all directions in the affected eye. No increase in the proptosis was noted with Valsalva maneuver and no bruit on auscultation was audible. The cornea and lens were clear. Choroidal folds were seen on fundus examination with normal disc and macula. Ultrasound showed multiple cystic intraconal spaces [Figure 2]. Computed tomography (CT) scan showed multiple hypodense non-enhancing intraconal lobulated lesions, extending into the preseptal space [Figure 3]. Contrast magnetic resonance imaging (MRI) of the orbit and brain demonstrated cystic morphology with no extension into the orbital apex or superior orbital fissure [Figure 4a,b]. No intracranial vascular lesion was detected. Subtotal excision of the mass was done through transconjunctival approach by using carbon dioxide laser ablation. Histopathologic examination revealed lymphoid tissue with large vascular channels, a few containing blood and organized thrombi, extensive hemosiderin deposition and trace amounts of fibrin. The patient was discharged in good general condition with marked improvement in proptosis and vision.

**Figure 4(A):**
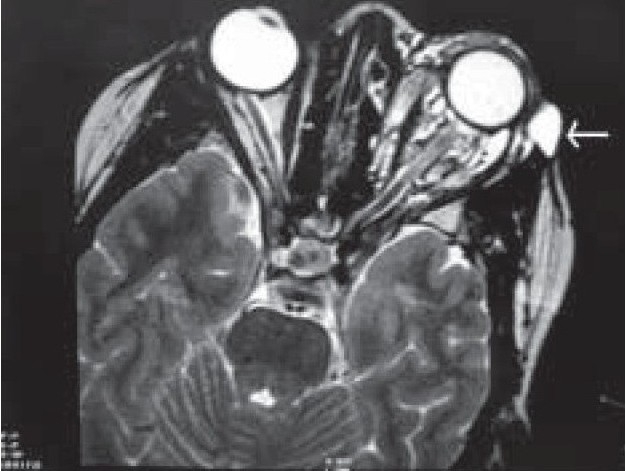
T2-axial MRI image shows extraconal extension (Arrow)

**Figure 4(B):**
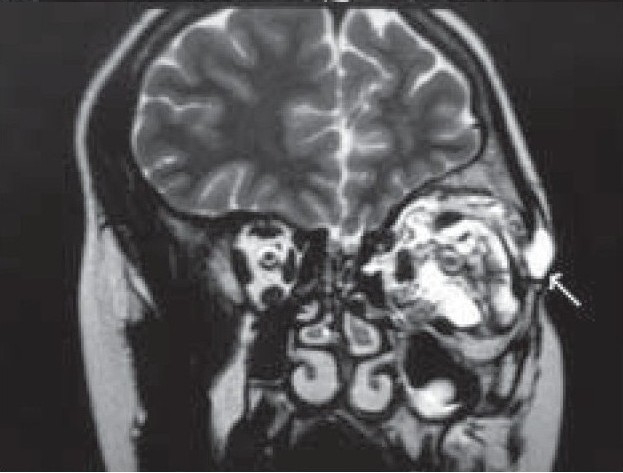
T2-coronal MRI section showing retrobulbar (intraconal) cystic spaces with extraconal extension (white arrow)

Systemic corticosteroids have been used as an adjuvant treatment to surgery, although their role is controversial. The diffuse form of orbital lymphangioma is well known for its difficult surgical treatment and frequent recurrences. Small, repeated excisions may be required. Surgical debulking with carbon dioxide laser through a lateral orbitotomy combined with three-wall orbital decompression is a preferred surgical technique and may be a useful alternative treatment in patients with severe proptosis.

Ultrasonography of OVLMs show high amplitude echoes with a very wide separation due to large encapsulated fluid lakes. On CT scan, these are poorly defined slightly enhancing lesions that cross anatomic boundaries, such as the conal fascia and orbital septum. Areas of hemorrhage cause cyst-like masses with rim enhancement. In the last decade, role of MRI has been emphasized in the literature as it has the capability to precisely delineate and characterize these lesions.[1] It is recommended to use surface coils for higher spatial resolution as this can differentiate between the typical vascular tumors. Song GX in 1991 compared ultrasound, CT and MRI in diagnosis of orbital disorders and concluded that MRI was superior in contrast resolution and spatial localization.[4] However, OVLMs may often have atypical features and MRI findings may not be characteristic.[5]

We conclude that imaging plays a crucial role in diagnosis of OVLMs and intracranial associations. However, atypical characteristics may be seen in a few pediatric patients and excision biopsy may be indicated to facilitate diagnosis.
